# A case of dupable duple duplicity and duplexity

**Published:** 2013

**Authors:** Uzma Afzal, Rasha Mater Al-Shammari, Qaisar H Siraj, Santosh Hebbar

**Affiliations:** 1 Department of Nuclear Medicine and Radiology, Farwania Hospital, Kuwait; 2 Bneid El-Qar Primary Health Care Center, Kuwait

**Keywords:** Vesicoureteric reflux, Horseshoe kidney, Ureteral duplication, Ureterocele

## Abstract

Duplication anomalies are quite common with ureteral duplication anomalies being the most frequent. Despite the relatively frequent incidence of a horseshoe kidney and duplication anomalies in any individual patient, the combination of horseshoe kidney and bilateral ureteric duplication is a very rare entity and very few cases have been reported to date. We present a case of a patient with a novel combination of a horseshoe kidney and multiple rare congenital renal anomalies and their sequelae.

## Introduction

Horseshoe kidney is the most common congenital renal and one of the commonest fusion anomalies (1, 2). The affected individuals may either be asymptomatic or may present with hydronephrosis, renal calculi and vesicoureteric reflux (VUR) (2). Horseshoe kidney may occur as an isolated entity but approximately one-third of the cases are associated with other congenital anomalies (3). Duplication anomalies are relatively common with ureteral duplication anomalies being the commonest (4). Ureteral duplication anomalies may present with urinary tract infection (UTI), as duplicated ureters are often associated with an obstructed upper pole moiety and a refluxing lower pole moiety (4).

Despite the relatively frequent incidence of a horseshoe kidney and duplication anomalies, the combination of horseshoe kidney and ureteric duplication is a very rare combination and very few cases have been reported to date (2, 3). We present a patient with a horseshoe kidney with bilateral double collecting systems, bilateral duplicated ureters and duplicated renal vessels, who had all the possible associated sequelae including UTI, left sided hydronephrosis, hydroureter, renal calculi, left ureterocoele and grade IV vesicoureteric reflux. This case appears to be a rare and novel combination of congenital renal anomalies and abnormalities.

## Case Report

A 30 year old female with a history of recurrent febrile UTIs since her childhood was incidentally diagnosed during an obstetric ultrasound with a horseshoe kidney and bilateral double collecting systems and duplication of ureters. She had a past medical history of left sided ureterocele treated 9 months ago with laparoscopic decompression. After a 3-month period of recurrent UTIs, the patient was referred to the nuclear medicine department for a ^99m^Tc-MAG3 (mercaptoac-etyltriglycine) renal scan for the assessment of renal function and to rule out obstruction and VUR.

**Figure 1:**
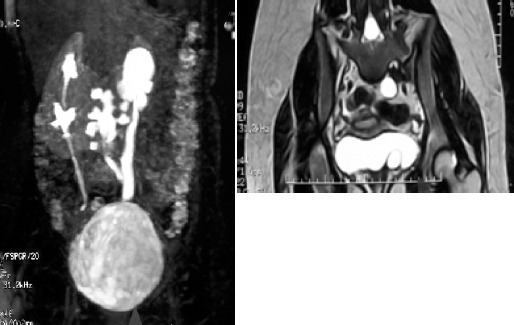
MRI showing bilateral duplex collecting system with double ureter and ureterocoele on the left.

**Figure 2:**
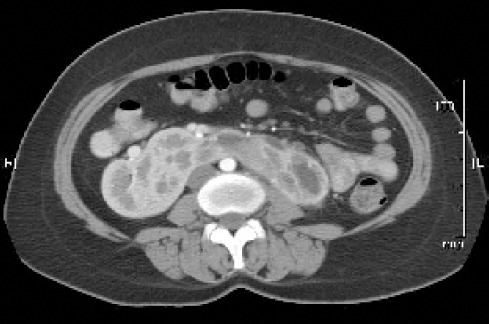
CT images showing lower moieties of both kidneys and the adjoining isthmus.

**Figure 3:**
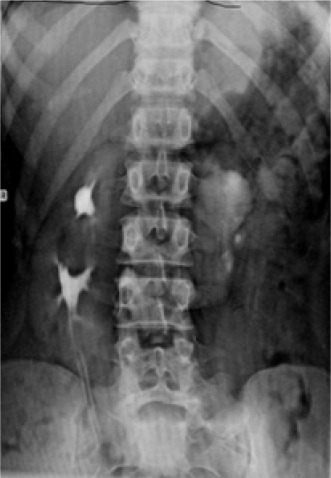
IVU showing double ureter on the right side.

Structural imaging included abdominal ultrasound, CT urogram, MRI and IVU (intravenous urography) which revealed a horseshoe kidney with bilateral duplex collecting systems, bilateral duplication of the ureters, duplicated renal arteries, bilateral tiny calculi, moderate left upper moiety hydronephrosis and significant hydroureter (Figures 1-3).

**Figure 4:**
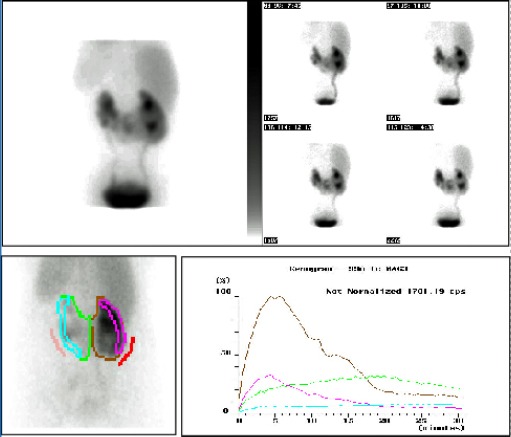
MAG3 renogram showing a normally functioning right kidney with no evidence of obstruction or VUR and relatively reduced functioning both moieties on the left without any evidence of obstruction.

MCUG (micturating cystouretherogram) showed left-sided grade IV VUR. The MAG3 renogram with Lasix washout and indirect cystography, showed a normally functioning right kidney with no evidence of obstruction or VUR; however, both moieties on the left had relatively reduced function without any evidence of obstruction, with significant VUR seen in the upper moiety of this kidney (Figure 4).

## Discussion

Renal fusion anomalies and duplication anomalies frequently occur independently or in association with other congenital anomalies; however, their occurrence together seems to be truly a rare event (1).

Duplication of the renal collecting system is the most common upper tract anomaly with an incidence of 0.5-0.8% and partial or complete duplication of the ureter has an estimated incidence of 0.8% (1). The combination of horseshoe kidney and bilateral ureteral duplication is an extremely rare entity. However, unilateral ureteral duplication is six times more frequent than bilateral duplication, with an equal chance of occurring on either side (2, 3). Only three cases of horseshoe kidney with bilateral ureteral duplication have been reported to date (2-4). Christoffersen *et al* had described partial hydronephrosis, bilateral duplication of the pelvis and the ureter with horseshoe kidney (2). Keskin et al reported a horseshoe kidney, bilateral double collecting systems and bilateral partial ureteral duplication (3). Kuzel et al described a horseshoe kidney with bilateral pelvic duplication and double ureters (4). Kevin *et al* reported a case of a horseshoe kidney with duplicated renal vessels and upper collecting systems in a middle-aged male cadaver (5).

**Figure 5:**
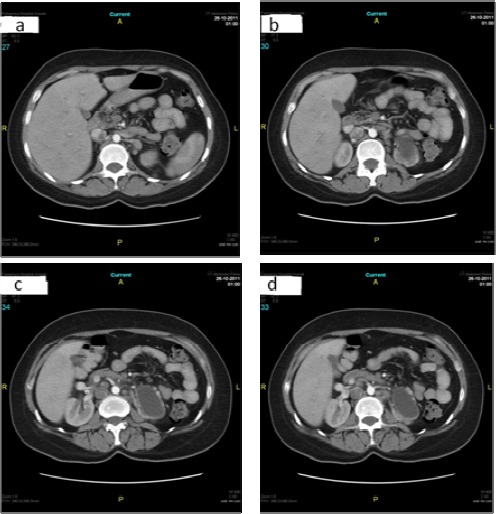
(a, b, c, d). Axial post-contrast arterial phase images (a, b& c) showing the origin of three right renal arteries from aorta and (a & d) showing two left renal arteries arising from the aorta.

The combination of a horseshoe kidney with bilateral double collecting systems, bilateral ureteral duplication and bilateral duplicated renal vessels with multiple concomitant abnormalities (including hydronephrosis, hydroureter, urolithiasis, vesicoureteric reflux and ureterocele) in a single patient, hasn’t been previously reported. The occurrence of unilateral or bilateral duplex systems in horseshoe kidneys may pose diagnostic and interventional challenges. Comprehensive structural and functional imaging is mandatory for ascertaining the precise anatomy and function of these kidneys prior to management or curative treatment when necessary.
